# Prevalence and mechanism of fluoroquinolone resistance in clinical isolates of *Proteus mirabilis* in Japan

**DOI:** 10.1016/j.heliyon.2019.e01291

**Published:** 2019-03-02

**Authors:** Ryuichi Nakano, Akiyo Nakano, Michiko Abe, Noriyuki Nagano, Miwa Asahara, Taiji Furukawa, Yasuo Ono, Hisakazu Yano, Ryoichi Okamoto

**Affiliations:** a Department of Microbiology and Infectious Diseases, Nara Medical University, Kashihara, Nara, Japan; b Department of Medical Laboratory Sciences, Kitasato University School of Allied Health Sciences, Sagamihara, Kanagawa, Japan; c Department of Health and Medical Sciences, Shinshu University, Graduate School of Medicine, Matsumoto, Nagano, Japan; d Department of Central Clinical Laboratory, Teikyo University Hospital, Itabashi-Ku, Tokyo, Japan; e Department of Microbiology and Immunology, Teikyo University School of Medicine, Itabashi-Ku, Tokyo, Japan; f Department of Microbiology, Kitasato University School of Medicine, Sagamihara, Kanagawa, Japan

**Keywords:** Microbiology, Epidemiology

## Abstract

Fluoroquinolone (FQ) and cephalosporin (CEP) resistance among Enterobacteriaceae has been increasingly reported. FQ resistance occurs primarily through mutations in DNA gyrase (*gyrA* and *gyrB*) and topoisomerase IV (*parC* and *parE*). CEP resistance in Enterobacteriaceae is mainly due to the production of CTX-M type extended-spectrum β-lactamases. Although prevalence and mechanisms of FQ and CEP resistance in Enterobacteriaceae such as *Escherichia coli* have been well studied, little is known about *Proteus mirabilis* in Japan. In this study, we assessed the prevalence and mechanism of FQ resistance in Japanese clinical isolates of *P. mirabilis*. We collected 5845 *P. mirabilis* isolates from eight hospitals between 2000 and 2013. Prevalence of FQ resistance was calculated as the annual average percentage of all *P. mirabilis* isolates. We selected 50 isolates exhibiting susceptibility, intermediate resistance, or resistance to levofloxacin (LVX) and identified amino acid substitutions in GyrA, GyrB, ParC, and ParE. The prevalence of FQ-resistant *P. mirabilis* gradually increased from 2001 to 2004, reaching 16.6% in 2005, and has remained relatively high (13.3–17.5%) since then. Low-level LVX-resistant strains (MIC, 8–16 mg/L) showed significant changes in GyrB (S464Y or -I, or E466D). High-level LVX-resistant strains (MIC, 32–128 mg/L) displayed significant changes in GyrA (E87K) and ParE (D420N). The highest-level LVX-resistant strains (MIC, ≥ 256 mg/L) presented significant changes in GyrA (E87K or -G), GyrB (S464I or -F), and ParE (D420N). Our findings suggest that substitutions in GyrA (E87) and ParE (D420) have played an important role in the emergence of high-level LVX-resistant *P. mirabilis* isolates (MIC, ≥ 32 mg/L) in Japan.

## 1 Introduction

*Proteus mirabilis* is one of the most common causative agents of urinary tract infections (UTIs). It can lead to pneumonia in debilitated or immunocompromised patients, and represents an important cause of nosocomial infections [1]. Fluoroquinolones (FQs), cephalosporins (CEPs), and co-trimoxazole are used as first-line agents against cystitis [2]. FQs are the most commonly used antibiotics for the treatment of a wide range of infections including UTIs in western Europe, North America, and Japan [3, 4]. As a result of their widespread use, rates of FQ- and CEP-resistant Enterobacteriaceae have increased worldwide [5, 6, 7, 8]. Data from the European Antimicrobial Resistance Surveillance Network confirmed a significant increase in FQ resistance across Europe since 2001 [9]. Furthermore, the prevalence of CEP-resistant *Escherichia coli* has also increased worldwide due to the production of extended-spectrum β-lactamases (ESBLs), particularly CTX-M enzymes [6, 10]. Although *P. mirabilis* wild-type strains are usually susceptible to FQs, increased resistance has been observed in clinical isolates [2, 11, 12, 13, 14]. In addition, we previously reported a regional outbreak of CTX-M-2 enzyme-producing *P. mirabilis* in Japan [15].

The mechanisms of resistance to FQs identified so far in clinical isolates include mainly alterations of target proteins, such as DNA gyrase (encoded by *gyrA* and *gyrB*) and topoisomerase IV (encoded by *parC* and *parE*), and decreased drug accumulation due to efflux pumps or changes in outer membrane [16, 17]. The primary target of FQs in several species of Enterobacteriaceae such as *E. coli* is GyrA. Specific point mutations in *gyrA* are associated with resistance to FQs [16, 18]. Additional mutations in DNA gyrase or topoisomerase IV contribute to high-level forms of resistance [19, 20]. Genetic characterisation of these mutations has been defined by DNA sequence analysis of clinical isolates; accordingly, highly conserved regions are designated as quinolone resistance-determining regions (QRDRs) [16, 21]. QRDRs associated with resistance to FQs in *P. mirabilis* include mostly known substitutions in GyrA (S83) and ParC (S80) [22, 23]. Additional mutations in GyrB (S464) confer high-level FQ resistance [22, 23]. However, the exact role of QRDRs, especially those on ParE, in FQ-resistant *P. mirabilis* is unclear and more data from clinical isolates are required to identify the mechanism of resistance.

Although the prevalence and mechanisms of FQ resistance are well established in *E. coli*, little is known about *P. mirabilis*. Hence, in this study, we carried out a survey to assess the prevalence and mechanisms of resistance to FQs among *P. mirabilis* clinical isolates in Japan between 2000 and 2013. Amino acid sequences of the QRDRs on GyrA, GyrB, ParC, and ParE from clinical isolates were compared to the type strain and susceptible isolates. In addition, amino acid substitution profiles were studied to determine associations between QRDR mutations, FQ resistance, and levofloxacin (LVX) minimum inhibitory concentrations (MICs).

## 2 Materials and methods

### 2.1 Epidemiological study of drug-resistant *P. mirabilis*

*P. mirabilis* isolates were collected from eight hospitals located in different Japanese cities between 2000 and 2013. The bed capacity of these hospitals exceeded 300 units. All strains were isolated from either infections or colonization/screening and only one isolate per patient was included in the study. The isolates were identified and initial susceptibilities of FQs and CEPs were determined using the MicroScan WalkAway System (Beckman Coulter, Inc.). The annual prevalence of resistance to FQs or CEPs was calculated as the annual average percentage of all *P. mirabilis* isolates resistant to LVX or cefotaxime (CTX). Resistance to LVX or CTX was defined by a MIC ≥8 mg/L or ≥4 mg/L, respectively [24].

### 2.2 Bacterial strains and determination of antimicrobial susceptibility

A total of 100 non-duplicate isolates were selected from the eight hospitals within different periods during 2004–2005. Antimicrobial susceptibility to the following drugs was determined by the agar dilution method according to the Clinical Laboratory Standards Institute guidelines using *E. coli* ATCC 25922 as a quality control strain [24]: nalidixic acid (NAL), LVX, ciprofloxacin (CIP), norfloxacin (NOR), ofloxacin (OFX), sparfloxacin (SPX), and sitafloxacin (STX). To evaluate the contribution of efflux pumps to FQ resistance, MICs were measured in the presence of the efflux pump inhibitor carbonyl cyanide m-chlorophenylhydrazone (CCCP). CCCP was incorporated in Mueller–Hinton agar at a concentration of 12.5 μM. Fifty isolates, whose FQ susceptibility was not affected by CCCP, were selected among 100 isolates and were further characterised in terms of genotype and MICs for LVX.

### 2.3 PCR amplification and sequencing of *gyrA*, *gyrB*, *parC*, and *parE* QRDRs

Twenty-three isolates showing susceptibility or intermediate resistance to LVX (MIC, ≤ 4 mg/L), and 27 LVX-resistant (MIC, ≥ 8 mg/L) *P. mirabilis* isolates were analysed for QRDR mutations in *gyrA*, *gyrB*, *parC,* and *parE* using PCR amplification and direct DNA sequencing. Oligonucleotide primers for PCR amplification of *P. mirabilis* ATCC29906 type strain genes were designed based on the alignment of the following known DNA sequences (accession numbers) in the GenBank database: AF397169 (*gyrA*), AF503506 (*gyrB*), AF363611 (*parC*), and AF503505 (*parE*). The primers were as follows: *gyrA*, forward, 5′-ACTGAAGCCAGTACACCG-3′, reverse, 5′-GATCTTCGCCATACGAAC-3′; *gyrB*, forward, 5′-GGTAGAAACGCTGATGAATG-3′, reverse, 5′-ATCTGGGTTATATTCATCACG-3′; *parC*, forward, 5′-ACAGCGTCGTATCGTCTAT-3′, reverse, 5′-CAGGGTGCCATCAAAAT-3′; *parE*, forward, 5′-CAAAGAGCGTCTATCGTCAC-3′, reverse, 5′-TCCGTAACGTAATTGGCTT-3′. Cycling conditions were as follows: denaturation at 94 °C for 10 min, followed by 35 amplification cycles (94 °C for 30 s, 54 °C for 30 s, 72 °C for 30 s), and a final elongation step at 72 °C for 5 min. DNA sequences were analysed and compared to *P. mirabilis* ATCC29906 using the NCBI BLAST tool. The presence of plasmid-mediated quinolone resistance *qnr* genes (*qnrA*, *qnrB*, *qnrC*, *qnrD*, and *qnrS*), *qepA*, and *aac(6′)-Ib-cr* was determined by PCR amplification [25, 26].

## 3 Results and discussion

### 3.1 Prevalence of resistance to LVX, CTX, or both in *P. mirabilis* isolates collected between 2000 and 2013

**Fig. 1:**
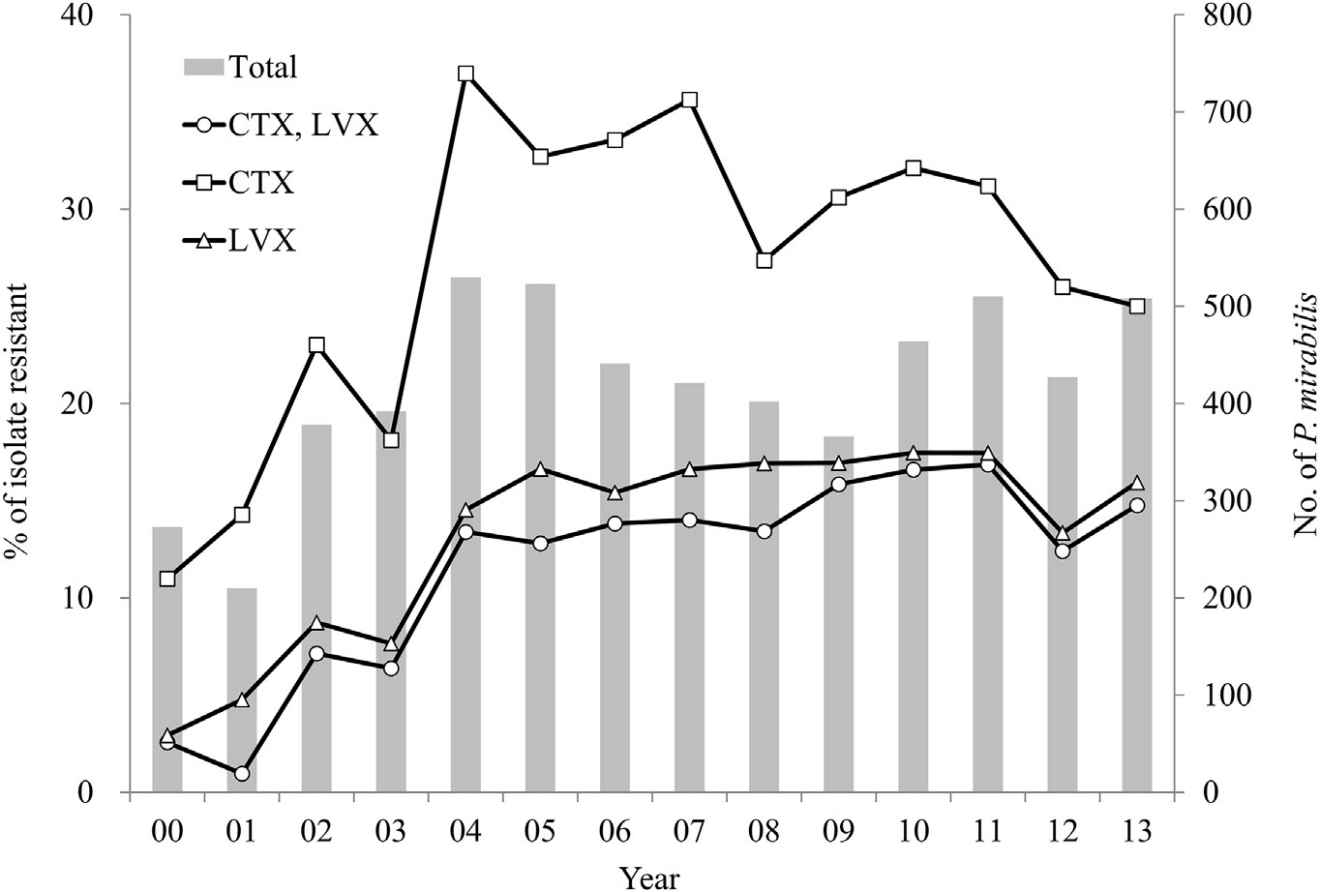
Prevalence of resistance to levofloxacin (LVX), cefotaxime (CTX), or both LVX and CTX in *P. mirabilis* isolates from Japan: ■, total number of *P. mirabilis* isolates per year; ○, % of CTX- and LVX-resistant *P. mirabilis* isolates per year; □, % of CTX-resistant *P. mirabilis* isolates per year; △, % of LVX-resistant *P. mirabilis* isolates per year. Isolates resistant to LVX presented a slightly higher rate than those resistant to both LVX and CTX. Most LVX-resistant isolates were also resistant to CTX. The histogram is linked to the 2^nd^ Y-axis representing the number of *P. mirabilis* isolates.

We conducted a 14-year survey of FQ resistance by screening 5845 clinical isolates of *P. mirabilis*. The frequency of *P. mirabilis* LVX- and/or CTX-resistant isolates for each year is shown in Fig. 1. It reveals a gradual increase in the rate of LVX resistance (MIC, ≥ 8 mg/L) between 2000 and 2005, and a sustained high prevalence of FQ-resistant *P. mirabilis* isolates (13.3–17.5%) since 2004. This trend reflects what was described previously in Japan and Europe [2, 27]. It also reveals that the prevalence of CTX-resistant *P. mirabilis* increased gradually, reaching 37.0% in 2004. Such high prevalence of CTX-resistant *P. mirabilis* isolates is similar to that reported previously in Japan between 2009 and 2010 (45.6%) [14]. We previously reported that almost all CEP-resistant *P. mirabilis* in Japan harboured a plasmid-borne *bla*_CTX-M-2_ gene [15]. The prevalence of *P. mirabilis* resistant to both LVX and CTX also increased. Our data indicate that 87.9 % of FQ-resistant isolates from the present 14-year survey were also resistant to CEP. These drug-resistant *P. mirabilis* are highly prevalent in Japan, where their frequency has remained almost unchanged since 2004.

This trend towards increased resistance to LVX is similar to that reported previously for hospital isolates of *E. coli* [28]. FQ resistance has been closely associated with ESBL production, which may derive from the prior heavy use of antibiotics [29, 30]. Both FQ- and CEP-resistant pathogens gain a survival advantage during antibiotic treatment with FQ or CEP. The resulting selective pressure favours these pathogens’ survival and spreading [6]. The worldwide dissemination of FQ- and CEP-resistant pathogens is a significant challenge to antibiotic treatment and infection control policies. *E. coli* is, like *P. mirabilis*, a common cause of UTIs and is also treated with FQs and CEPs. The widespread cumulative use of FQs may be accelerating the development of resistance to these agents, and may represent the driving force behind the stepwise increase in resistance among Enterobacteriaceae [31]. Whereas the percentage of *E. coli* isolates resistant to LVX has been higher than that of CEP-resistant strains [10], in the present study we observed the opposite trend for *P. mirabilis*.

### 3.2 Amino acid substitutions in GyrA, GyrB, ParC, and ParE QRDRs

Fifty *P. mirabilis* isolates and type strains with various levels of susceptibility to LVX (MICs, ≤ 0.063 to >512 mg/L) were selected by district genotype. Their *gyrA*, *gyrB*, *parC*, and *parE* genes were analysed to identify amino acid substitutions in the corresponding QRDRs. Gene amplification produced fragments of varying lengths: 271 bp for *gyrA*, 415 bp for *gyrB*, 331 bp for *parC*, and 451 bp for *parE*. These fragments corresponded to the following amino acids in *E. coli*: GyrA, 41 to 130; GyrB, 350 to 487; ParC, 42 to 151; and ParE, 334 to 483. In addition to LVX, we determined each strain's susceptibility profiles to CIP, NOR, OFX, SPX, and STX. Amino acid substitutions in the QRDRs of *gyrA*, *gyrB*, *parC*, and *parE* for each clinical isolate were compared to those of the ATCC 7002 type strain. Susceptibility profiles for LVX correlated with amino acid changes in QRDRs, as summarised in Table 1. All isolates were negative for plasmid-mediated quinolone resistance genes.

**Table 1:** Minimum inhibitory concentrations (MICs) of fluoroquinolones and associated substitutions in the quinolone resistance-determining regions of GyrA/B and ParC/E.

Strains (No. of strains)	MICs (mg/L)^a^							Amino acid substitutions in:^b^								
	NAL	LVX	CIP	NOR	OFX	SPX	STX	GyrA		GyrB		ParC		ParE		
								83	87	464	466	80	84	364	420	460
ATCC7002	8	≤0.063	≤0.063	0.125	0.25	0.25	≤0.063	S	E	S	E	S	E	V	D	E
(2)	4	≤0.063	≤0.063	≤0.063	≤0.063	0.125	≤0.063	-	-	-	-	-	-	-	-	-
(2)	8	0.125	≤0.063	0.25	≤0.063	0.25	≤0.063	I	-	-	-	I	-	-	-	-
(2)	16	0.25–1	0.5–1	0.25–0.5	2	2	0.125	I	-	-	-	-	-	I	-	K
(3)	8–16	0.25–0.5	0.5	0.25	2	1–2	0.125	I	-	-	-	I	-	I	-	K
(3)	>512	1	1–2	2	2–8	8–16	0.25	I	-	-	-	R	-	I	-	-
(11)	>512	1–4	2–8	2–8	8–16	8–16	0.25–0.5	I	-	-	-	I	-	I	-	-
(5)	>512	8–16	8–16	8–32	16	32–128	0.25–0.5	I	-	-	D	I	-	I	-	-
(3)	>512	8–16	8–16	8–32	16	32–128	1–2	I	-	Y	-	I	-	I	-	-
(2)	>512	8–16	8–16	8–32	16	32–128	1–2	I	-	Y	-	-	-	I	-	-
(2)	>512	8–16	8–16	8–32	32–64	16–64	0.5–1	I	-	I	-	I	D	I	-	-
MP10	>512	32	64	256	64	>512	0.5	I	-	-	D	I	K	I	-	-
TK1086	>512	32	32	64	64	>512	0.25	I	-	I	K	I	D	I	-	-
YP32	>512	32	8	32	8	32	2	I	-	-	-	-	-	I	N	-
(8)	>512	32–128	8–32	32–64	8–32	64–128	4–8	I	K	-	-	I	-	I	N	-
(2)	>512	256	512	512	512	128	32	I	K	I	-	F	-	-	N	-
(2)	>512	>512	512	256	512	512	16	I	G	F	-	I	-	-	N	-

a Abbreviations: NAL, nalidixic acid; LVX, levofloxacin; CIP, ciprofloxacin; NOR, norfloxacin; OFX, ofloxacin; SPX, sparfloxacin; STX, sitafloxacin.

b No change relative to type strains sequence.

For the purpose of data analysis, the isolates were subdivided into five groups according to LVX MICs and were then associated with QRDR substitutions: 1) equally supersensitive as the type strain (MIC, ≤ 0.063 mg/L); 2) sensitive and intermediate (MIC, 0.125–4 mg/L); 3) low-level resistance (MIC, 8–16 mg/L); 4) high-level resistance (MIC, 32–128 mg/L); and 5) highest-level resistance (MIC, ≥ 256 mg/L). LVX-wild type isolates revealed identical QRDR patterns as the type strain. LVX-susceptible and intermediate isolates with a LVX MIC of 0.125 mg/L presented substitutions in GyrA (S83I) and ParC (S80I); those with a MIC of 0,25–4 mg/L exhibited substitutions in ParE (V364I), together with single substitutions in GyrA (S83I) and ParC (S80). Low-level LVX-resistant strains displayed two additional substitutions in GyrB, S464 or E466. Substitutions in GyrB had a discernible influence on MICs, as previously reported [22, 27].

High-level LVX-resistant strains showed no substitutions in GyrB except for strains MP10 and TK1086, but displayed significant alterations in ParE (D420N). This substitution may have a discernible effect on the MIC, as shown by mutant YP32 (MIC, 32 mg/L). The additional substitution E87K in GyrA may further increase susceptibility (from 32 to 128 mg/L). Among strains with a LVX MIC of 32 mg/L, MP10 and TK1086 were super-resistant to SPX (MIC, > 512 mg/L). The MP10 strain had a substitution in ParC (E84K) in addition to substitutions from low-level resistant strains (such as GyrA S80I, GyrB E466D, ParC S80I, and ParE V364I). The TK1086 strain presented a substitution in GyrB (E466K) in addition to substitutions from low-level resistant strains (such as GyrA S83I, GyrB S464I, ParC S80I and E84D, and ParE V364I). These two strains’ extremely high resistance to SPX may depend on substrate specificity derived from the additional substitutions in GyrB (E466) and ParC (E84). The highest-level LVX-resistant strains showed changes in GyrA (S83I, E87K or -G), GyrB (S464I or -F), ParC (S80F or -I), and ParE (D420N). Substitutions in GyrA (E87G), GyrB (S464I or -F), and ParE (D420N) had a discernible effect on the MIC, and caused the highest-level of resistance to other FQs as well (except STX). A comparison of the inhibitory activities of FQs against DNA gyrase and topoisomerase IV in wild-type and QRDR-mutated strains will reveal the effect of FQ specificity on the selection of different enzyme substitutions [32].

For *P. mirabilis* isolates, LVX resistance arises in a stepwise fashion, with the accumulation of point mutations that result in amino acid substitutions within the QRDRs of DNA gyrase (*gyrA* and *gyrB*) and topoisomerase IV (*parC* and *parE*). Accordingly, low-level LVX-resistant strains (MIC, 8–16 mg/L) showed additional substitutions in GyrB (S464Y or –I, E466D); High-level resistant strains (MIC, 32–128 mg/L) presented additional substitution in both GyrB (E466D or -K) and ParC (E84K or -D), or ParE (D420N); the highest-level LVX-resistant strains (MIC, ≥ 256 mg/L) displayed additional substitutions in GyrA (E87G), GyrB (S464I or -F), and ParE (D420N). In contrast to *P. mirabilis*, many Enterobacteriaceae such as *E. coli* show an increase in FQ MICs following cumulative changes, mainly double mutations in GyrA and additional mutations in ParC [33]. Previous studies suggested that substitutions in GyrB (S464) and ParC (S80) might affect high-level FQ resistance. However, in *P. mirabilis*, the corresponding *E. coli* substitutions in GyrA (S83) and ParC (S80) did not significantly correlate with high-level resistance to FQs [22, 23]. In this study, substitutions in *P. mirabilis* GyrA (E87) and ParE (D420) had a significant effect on high-level resistance to FQs (MIC, ≥ 32 mg/L). *P. mirabilis* GyrA, GyrB, ParC, and ParE QRDRs have been compared to analogous sequences in *E. coli* [30], but showed low homology, particularly due to the high variability of ParE compared to ParC [30]. Such low homology could affect the correlation between QRDR mutations and FQ MICs, as this association forms the basis of the FQ-resistant mechanism in Enterobacteriaceae.

## 4 Conclusions

In conclusion, we conducted a survey of the prevalence of LVX-resistant *P. mirabilis* and investigated the mechanism of FQ resistance. In Japan, the prevalence of FQ-resistant *P. mirabilis* has gradually increased and has remained high (13.3–17.5%) since 2004. To tackle this problem, we must carefully monitor drug-resistant *P. mirabilis*, and limit and reduce the use of antibiotics. Our findings suggest that mutations in GyrA (E87) and ParE (D420) play an important role in the emergence of high-level FQ resistance in clinical isolates of *P. mirabilis*. This is the first report describing an association between the QRDRs of ParE and FQ resistance in *P. mirabilis*.

## Declarations

### Author contribution statement

Ryuichi Nakano, Akiyo Nakano: Conceived and designed the experiments; Performed the experiments; Analyzed and interpreted the data; Wrote the paper.

Michiko Abe, Noriyuki Nagano, Miwa Asahara: Analyzed and interpreted the data; Contributed reagents, materials, analysis tools or data.

Taiji Furukawa: Contributed reagents, materials, analysis tools or data.

Yasuo Ono, Hisakazu Yano, Ryoichi Okamoto: Conceived and designed the experiments.

### Funding statement

This work was supported by the Japan Society for the Promotion of Science (JSPS) KAKENHI (Grant no. 17K16228 and 17K10027).

### Competing interest statement

The authors declare no conflict of interest.

### Additional information

No additional information is available for this paper.
